# Supplementary material for: Prevalence of Variant GTRI Staphylococcus aureus Isolated from Dairy Cow Milk Samples in the Alpine Grazing System of the Aosta Valley and Its Association with AMR and Virulence Profiles
Source: Antibiotics (Basel). 2025 Mar 27;14(4):348. doi: 10.3390/antibiotics14040348 (PMC12024214; doi:10.3390/antibiotics14040348)
Supplement: Supplementary file 1 [file antibiotics-14-00348-s001.zip › antibiotics-3496399-supplementary.pdf]

Prevalence of Variant GTR[I] *Staphylococcus aureus* Isolated from Dairy Cow Milk Samples in the Alpine Grazing System of the Aosta Valley and Its Association with AMR and Virulence Profiles

Valentina Monistero [1,2,†], Delower Hossain [1,2,3,†], Sara Fusar Poli [1,2], Elizabeth Sampaio de Medeiros [1,2,4], Paola Cremonesi [5], Bianca Castiglioni [5], Filippo Biscarini [5], Hans Ulrich Graber [6], Giulia Mochettaz [7], Sandra Ganio [7], Alessandra Gazzola [8], Maria Filippa Addis [1,2], Claudio Roullet [7], Antonio Barberio [9], Silvia Deotto [9], Lara Biasio [9], Fernando Ulloa [10], Davide Galanti [1], Valerio Bronzo [1,2] and Paolo Moroni [1,2,*]

[1] Dipartimento di Medicina Veterinaria e Scienze Animali, Università Degli Studi di Milano, 26900 Lodi, Italy; valentina.monistero@unimi.it (V.M.); delower.hossain@unimi.it (D.H.); sara.fusar@unimi.it (S.F.P.); elizabeth.medeiros@ufrpe.br (E.S.d.M.); filippa.addis@unimi.it (M.F.A.); davide.galanti@unimi.it (D.G.); valerio.bronzo@unimi.it (V.B.)

[2] Laboratorio di Malattie Infettive Degli Animali-MiLab, University of Milan, 26900 Lodi, Italy

[3] Department of Medicine and Public Health, Faculty of Animal Science and Veterinary Medicine, Sher-e-Bangla Agricultural University (SAU), Dhaka 1207, Bangladesh

[4] Laboratório de Inspeção de Carne e Leite, Departamento de Medicina Veterinária, Universidade Federal Rural de Pernambuco (UFRPE), Recife 51171-900, PE, Brazil

[5] Institute of Agricultural Biology and Biotechnology, National Research Council, 26900 Lodi, Italy; paola.cremonesi@ibba.cnr.it (P.C.); bianca.castiglioni@ibba.cnr.it (B.C.); filippo.biscarini@ibba.cnr.it (F.B.)

[6] Food Microbial Systems, Microbiological Safety of Foods of Animal Origin Group, Agroscope, 3003 Bern, Switzerland; hans.graber-p@outlook.com

[7] Dipartimento di Prevenzione AUSL Della Valle d'Aosta, 11100 Aosta, Italy; giulia.mochettaz@libero.it (G.M.); sganio@ausl.vda.it (S.G.); croullet@ausl.vda.it (C.R.)

[8] Istituto Zooprofilattico Sperimentale Della Lombardia e Dell'emilia-Romagna, 26900 Lodi, Italy; alessandra.gazzola@izsler.it

[9] Istituto Zooprofilattico Sperimentale Delle Venezie, 35020 Legnaro, Italy; abarberio@izsvenezie.it (A.B.); sdeotto@izsvenezie.it (S.D.); lbiasio@izsvenezie.it (L.B.)

[10] Escuela de Graduados, Facultad de Ciencias Veterinarias, Universidad Austral de Chile, 5090000 Valdivia, Chile; fernando.ulloa@uach.cl

* Correspondence: paolo.moroni@unimi.it; Tel.: +39-02503-34584

† These authors contributed equally to this work.

Supplementary Materials

Figure S1. Distribution of genotypes of *S. aureus* at different timepoints (T1, T2 and T3) in different farms.

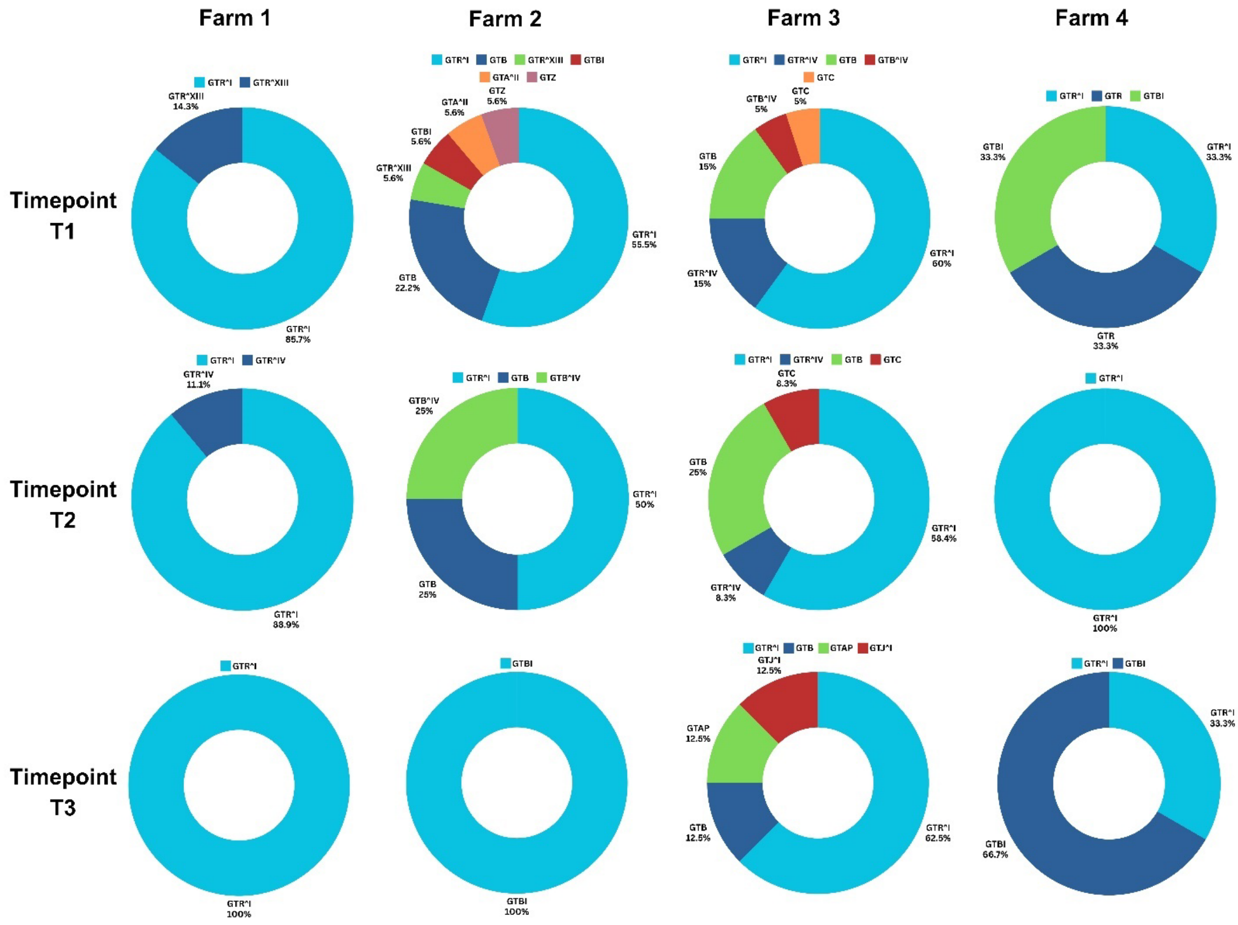

Table S1. Distribution of genotypes or variants (number of isolates) across the four mountain pastures at the three experimental timepoints (T1, T2, and T3). The most prevalent genotype in each mountain pasture is highlighted in **bold**.

Farms	Genotype Cluster (CL)	Genotype or variants	T1	T2	T3	Total
**1**	**CLR**	**GTR^{I}**	6	8	4	**18**
		GTR^{XIII}	1	-	-	1
		GTR^{IV}	-	1	-	1
						**20**
**2**	**CLR**	**GTR^{I}**	10	2	-	**12**
		GTR^{XIII}	1	-	-	1
	CLB	GTB	4	1	-	5
		GTB^{IV}	-	1	-	1
	CLBI	GTBI	1	-	1	2
	CLA	GTA^{II}	1	-	-	1
	CLZ	GTZ	1	-	-	1
						**23**
**3**	**CLR**	**GTR^{I}**	12	7	5	**24**
		GTR^{IV}	3	1	-	4
	CLB	GTB	3	3	1	7
		GTB^{IV}	1	-	-	1
	CLC	GTC	1	1	-	2
	CLAP	GTAP	-	-	1	1
	CLJ	GTJ^{I}	-	-	1	1
						**40**
**4**	**CLR**	**GTR^{I}**	2	1	1	**4**
		GTR	2	-	-	2
	CLBI	GTBI	2	-	2	4
						**10**

Table S2. Distribution of antimicrobial usage and number of treated cows across four dairy farms from 2021-2022

**Farms/Antibiotics used**	**Number of treated cows**		
	**2021**	**2022**	**Total**
**Farm 1**	**51**	**19**	**70**
(Longocillin LA) Amoxicillin	3	-	3
(Mastorange) Rifaximin	31	2	33
(Repen) Penicillins, in combination with other antibacterials	11	6	17
(Synulox Endomammary) Amoxicillin + clavulanate	6	11	17
**Farm 2**	**30**	**28**	**58**
(Cefatron) Cefapirin	3	-	3
(Ceftiocyl) Ceftiofur	1	-	1
(Ceftionil) Ceftiofur	4	-	4
(Cevaxel RTU) Ceftiofur	2	-	2
(Cobactan 25 Mg/Ml Soapension Injected) Cefquinome	1	-	1
(Fatroximin) Rifaximin	3	2	5
(Galactis) Lincomycin	-	2	2
(Mamyzin) Penethamate Hydriodide	-	2	2
(Mastivia) Cefquinome	2	-	2
(Mycospectone) Lincomycin, Combinations	10	12	22
(Oxtra -Injectable Solution) Oxytetracycline	-	2	2
(Repen) Penicillins, in combination with other antibacterials	1	2	3
(Rilexine 200 T) Cephalexin	-	1	1
(Synulox Endomammary) Amoxicillin + clavulanate	-	2	2
(Ubrolexin) Cefalexin, in combination with other antibacterials	3	3	6
**Farm 3**	**69**	**34**	**103**
(Axentyl 200 Mg/Ml Solution for Injection) Tylosin	-	2	2
(Cefatron) Cefapirin	43	9	52
(Ceftiocyl) Ceftiofur	5	-	5
(Mamyzin) Penethamate Hydriodide		2	2
(Mycospectone) Lincomycin, Combinations	16	6	22
(Oxtra -Injectable Solution) Oxytetracycline	-	6	6
(Repen) Penicillins, in combination with other antibacterials	5	6	11
(Standalone) Cefoperazone	-	3	3
**Farm 4 (A+B)**	**154**	**64**	**218**
(Ceftiocyl) Ceftiofur	2	-	2
(Cormicin Suspension Injected) Antibacterials and corticosteroids	-	6	6
(Fatroximin) Rifaximin	90	10	100
(Mastorange) Rifaximin	13	-	13
(Mycospectone) Lincomycin, combinations	18	14	32
(Oxtra -Injectable Solution) Oxytetracycline		1	1

Farms/Antibiotics used	Number of treated cows		
	2021	2022	Total
(Repen) Penicillins, in combination with other antibacterials	10	33	43
(Spirazine) Macrolides, in combination with other antibacterials	9	-	9
(Synulox-Suspension Injected) Amoxicillin + clavulanate	5	-	5
(Synulox Endomammary) Amoxicillin + clavulanate	6	-	6
(Ubrolexin) Cefalexin, in combination with other antibacterials	1	-	1
**Total**	**304**	**145**	**449**
